# Supplementary figures and images for: LINC00106/RPS19BP1/p53 axis promotes the proliferation and migration of human prostate cancer cells
Source: PeerJ. 2023 May 8;11:e15232. doi: 10.7717/peerj.15232 (PMC10174055; doi:10.7717/peerj.15232)

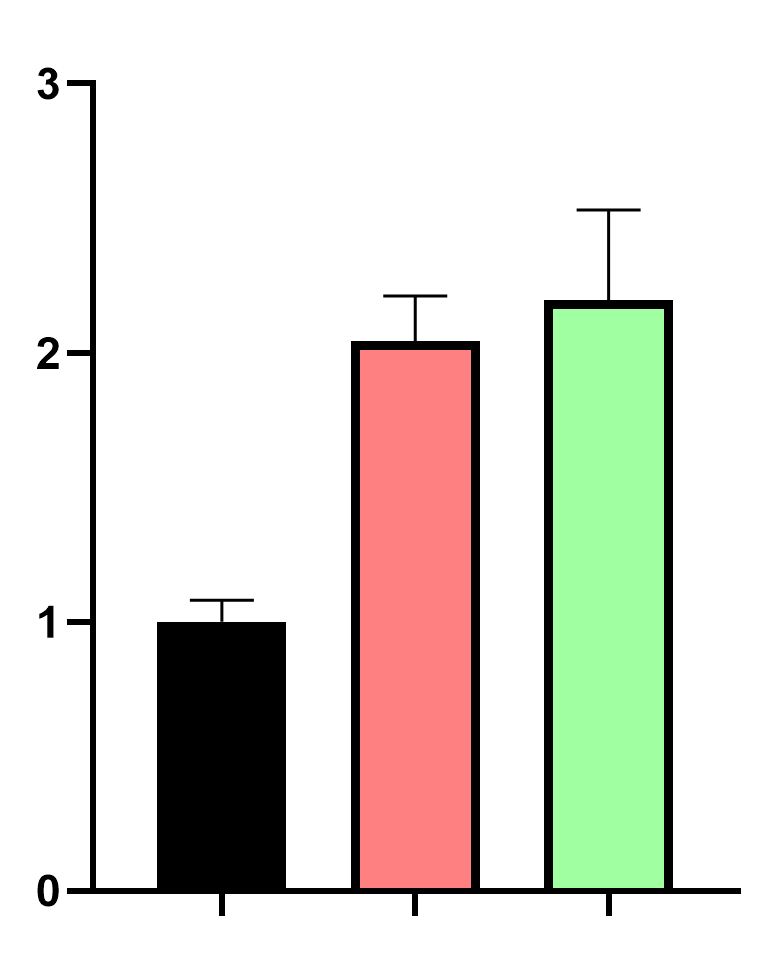

Supplement: Supplemental Information 1 [file peerj-11-15232-s001.zip › Luciferase Assay/Knock down LINC00106/du145.tif]

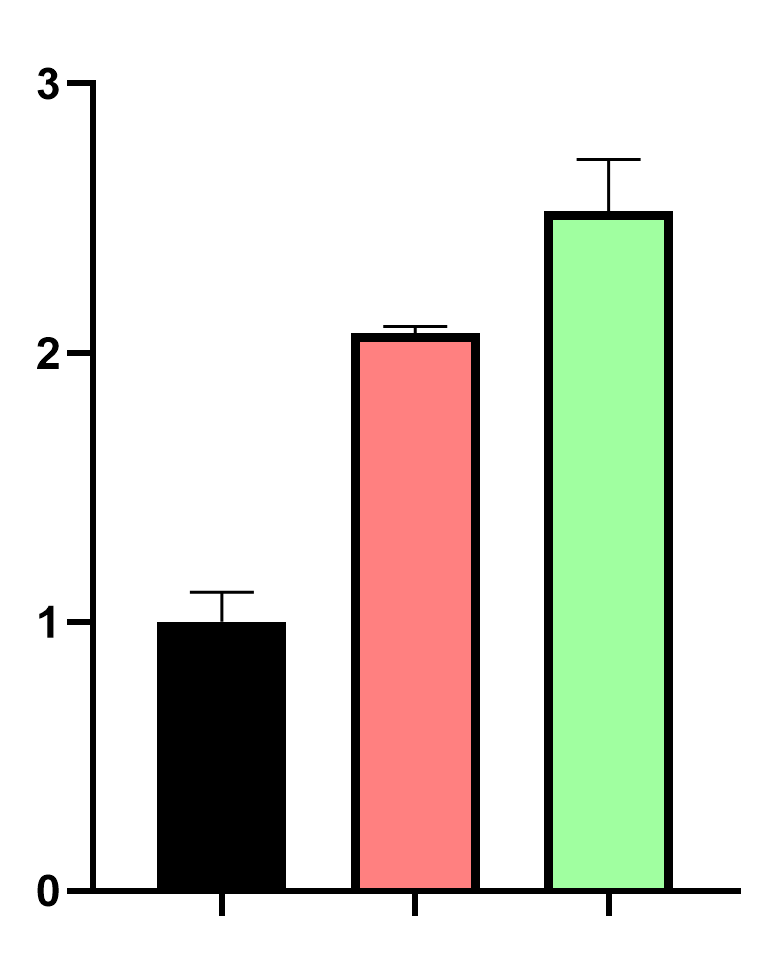

Supplement: Supplemental Information 1 [file peerj-11-15232-s001.zip › Luciferase Assay/Knock down LINC00106/pc3.tif]

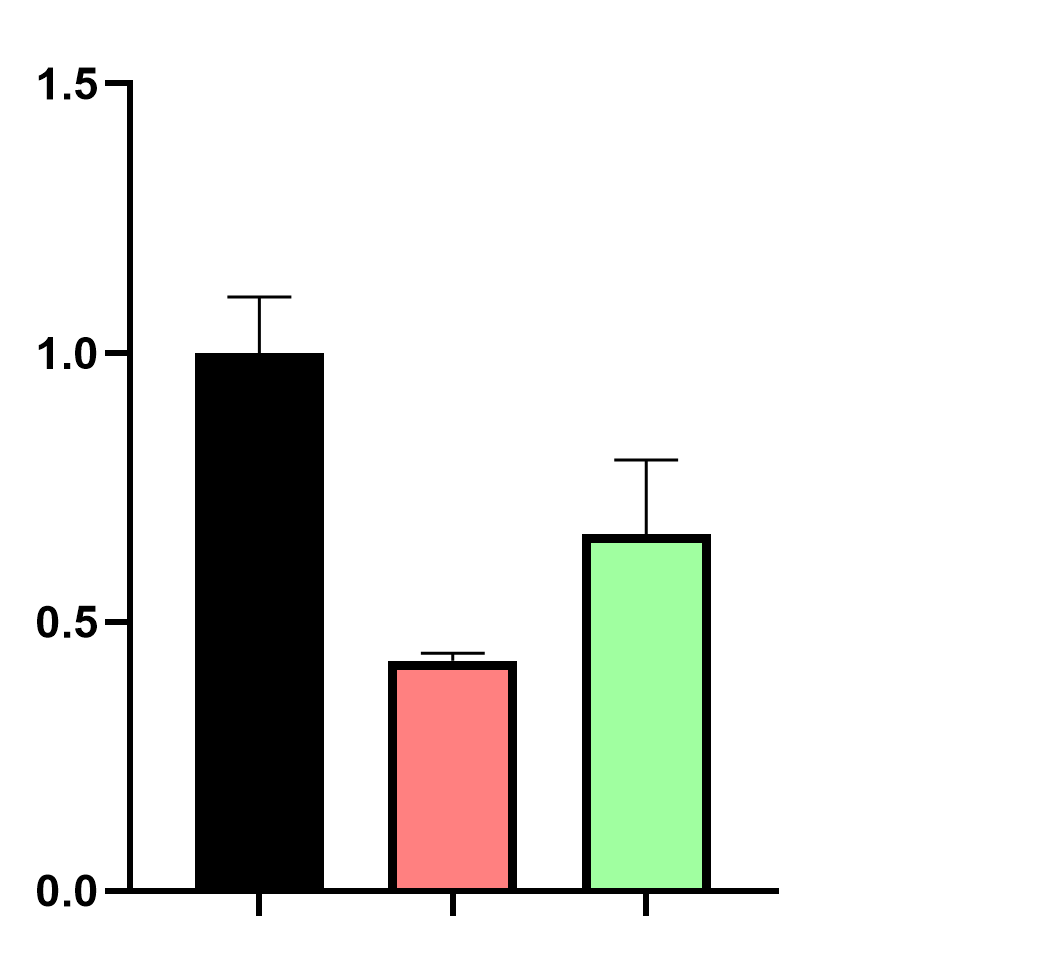

Supplement: Supplemental Information 1 [file peerj-11-15232-s001.zip › Luciferase Assay/Overexpression of RPS19BP1/du145.tif]

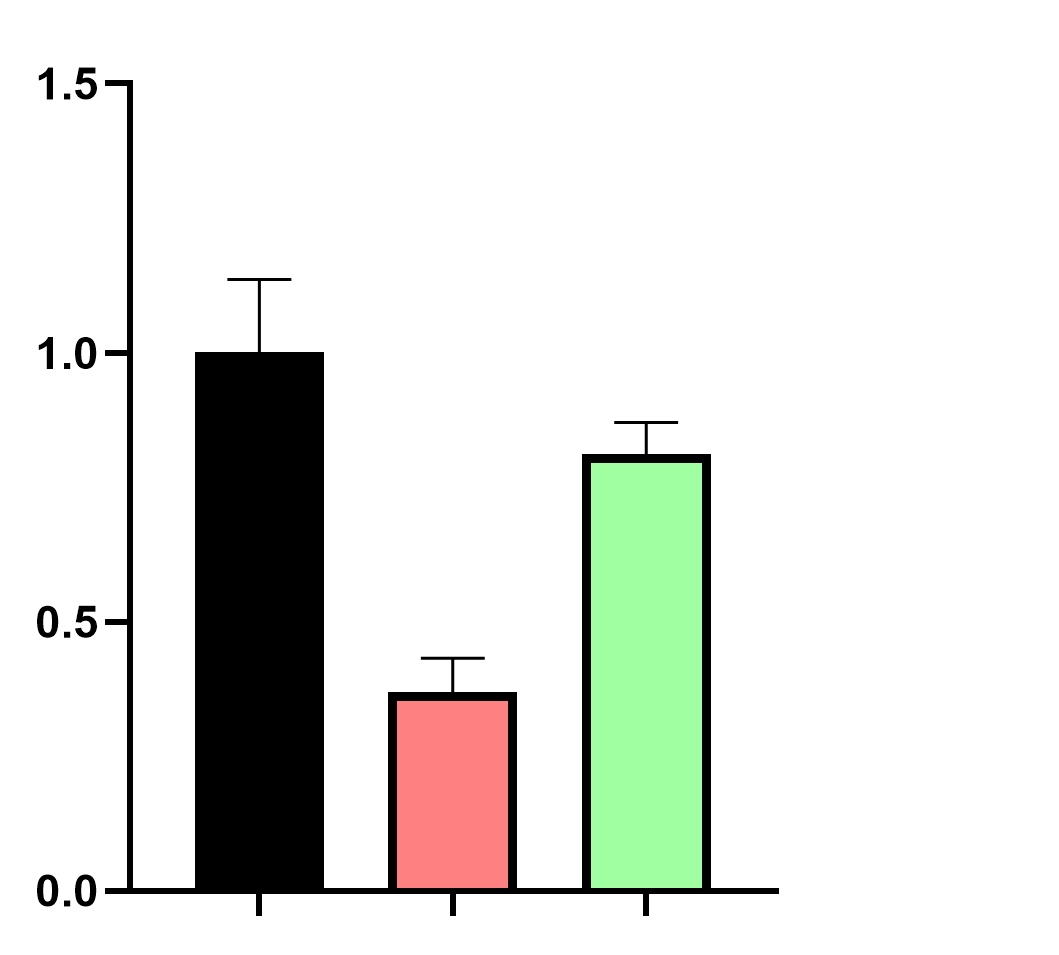

Supplement: Supplemental Information 1 [file peerj-11-15232-s001.zip › Luciferase Assay/Overexpression of RPS19BP1/pc3.tif]

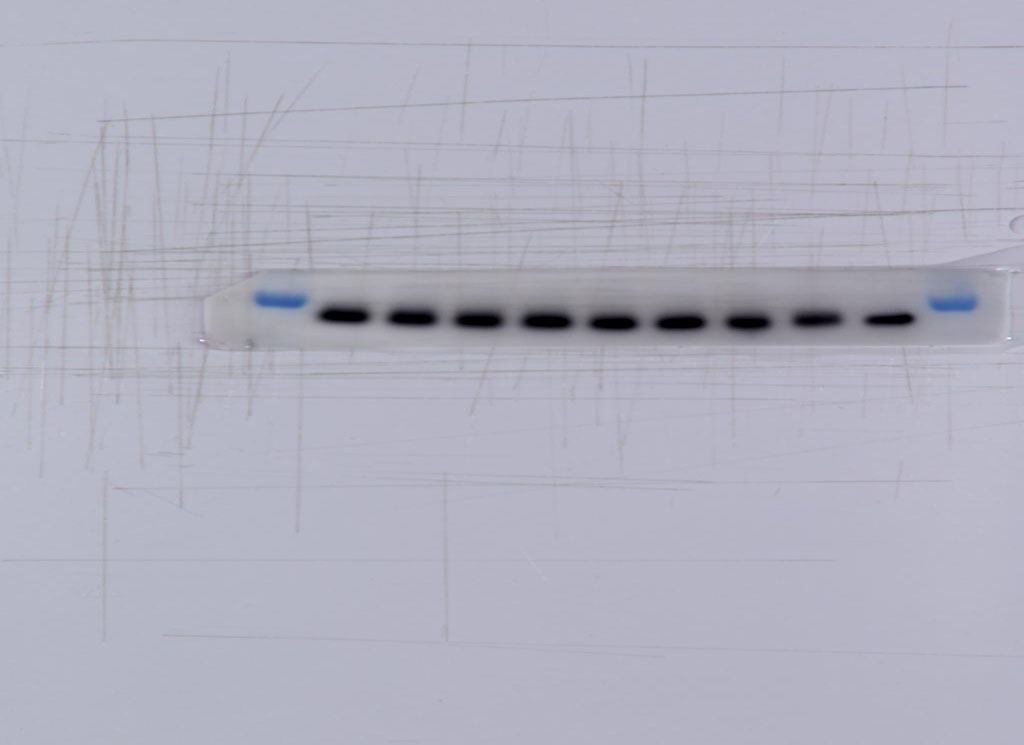

Supplement: Supplemental Information 3 [file peerj-11-15232-s003.zip › wb/P53/P53.1 2022.10.20_20.05.34_Ch+Marker.jpg]

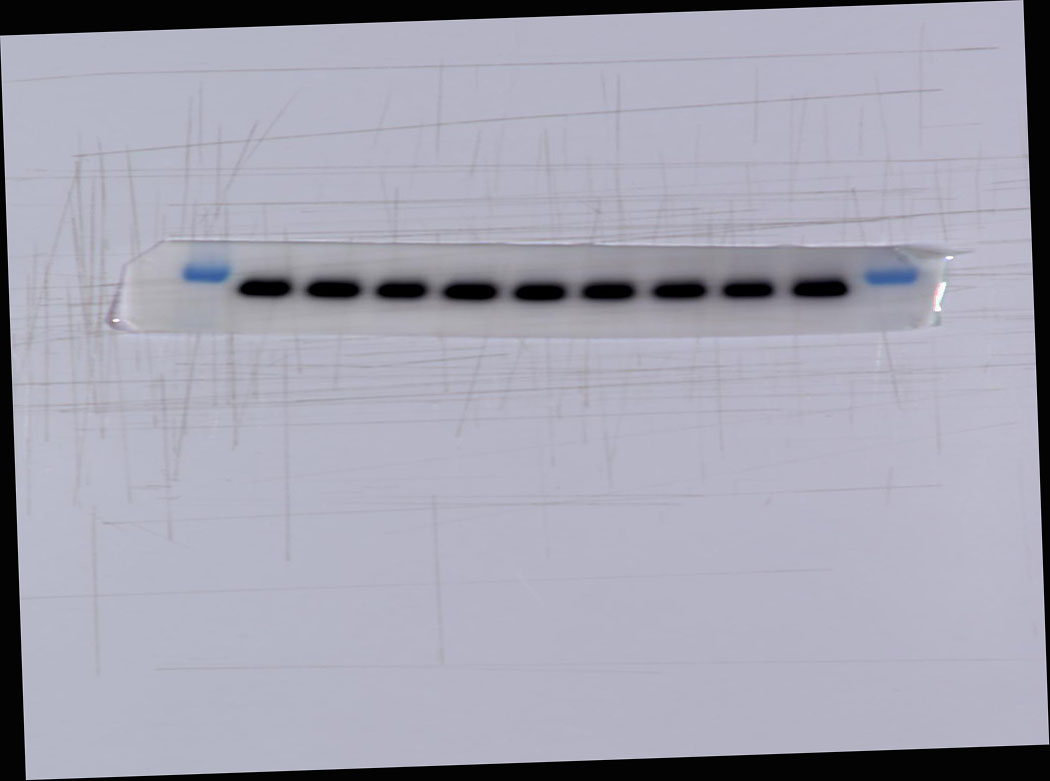

Supplement: Supplemental Information 3 [file peerj-11-15232-s003.zip › wb/P53/P53.2 2022.10.20_20.07.18_Ch+Marker.jpg]

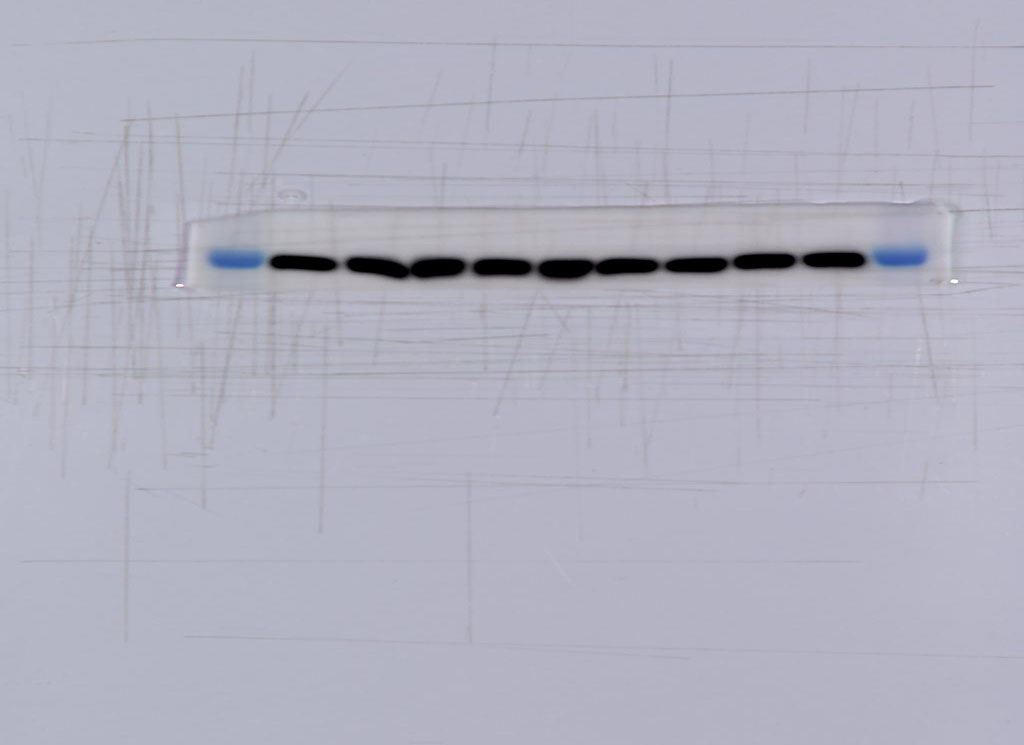

Supplement: Supplemental Information 3 [file peerj-11-15232-s003.zip › wb/P53/tubulin1 2022.10.20_14.59.59_Ch+Marker.jpg]

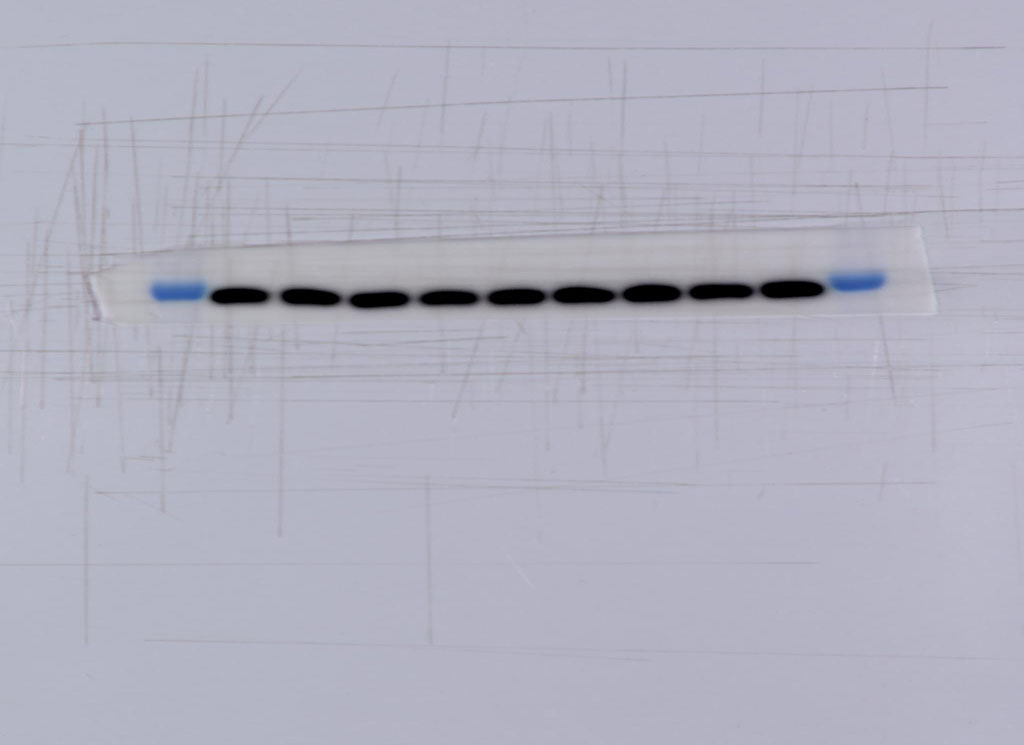

Supplement: Supplemental Information 3 [file peerj-11-15232-s003.zip › wb/P53/tubulin2 2022.10.20_15.04.09_Ch+Marker.jpg]

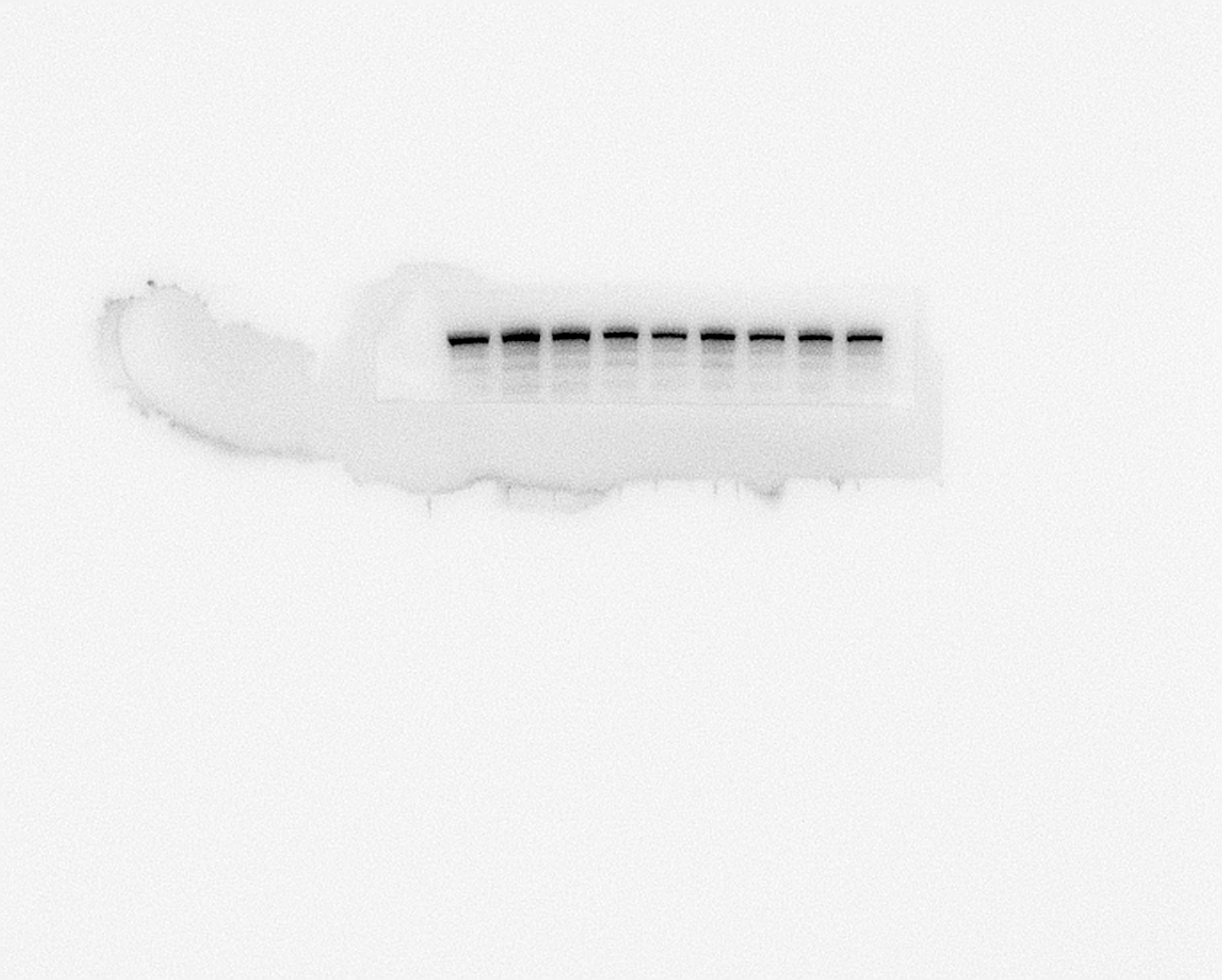

Supplement: Supplemental Information 3 [file peerj-11-15232-s003.zip › wb/RPS║═LINC/RPS19BP1▓╗▒Σ pc-3/10s-│÷═╝╙├.Tif]

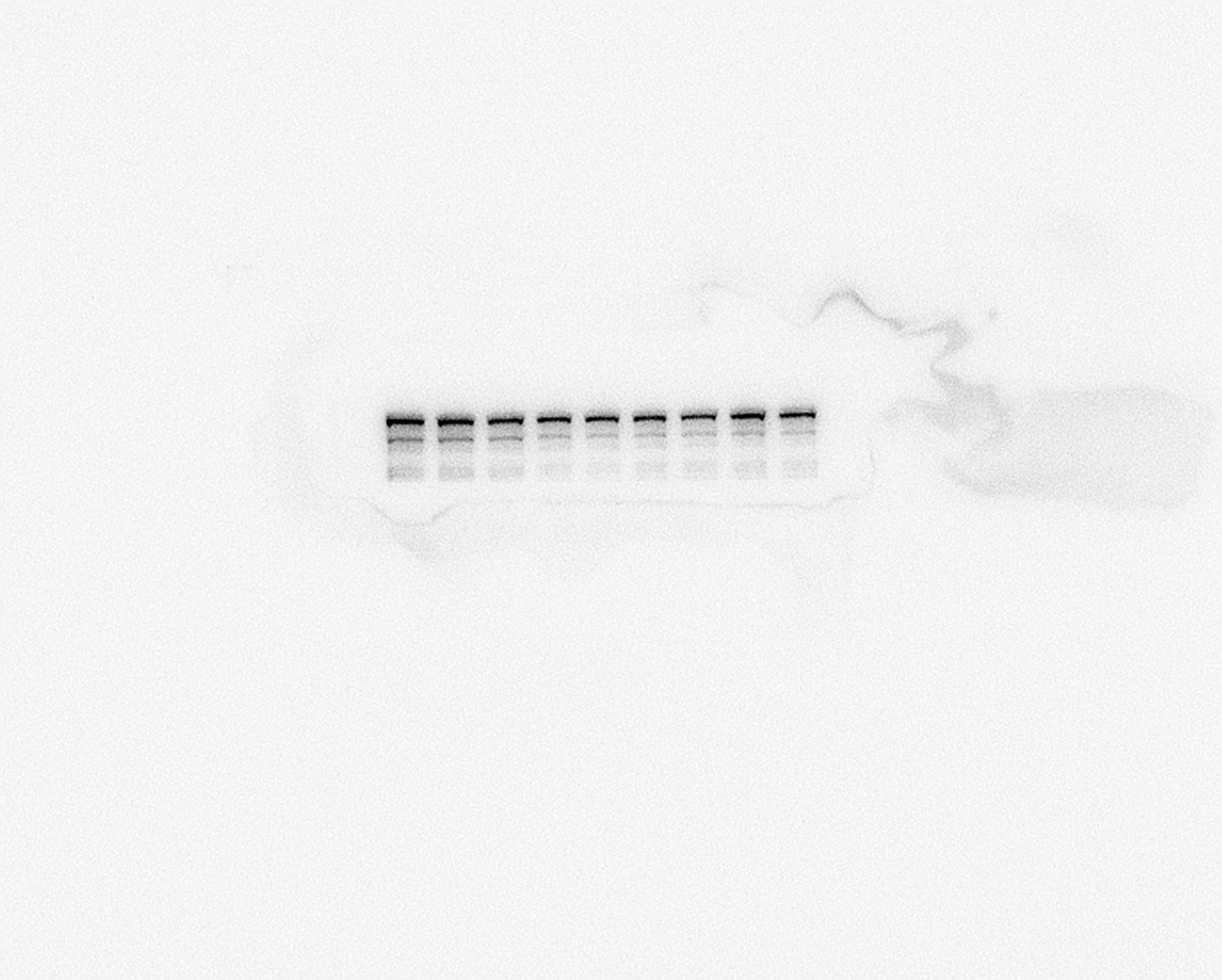

Supplement: Supplemental Information 3 [file peerj-11-15232-s003.zip › wb/RPS║═LINC/RPS19BP1▓╗▒Σdu145/8s-│÷═╝╙├.Tif]

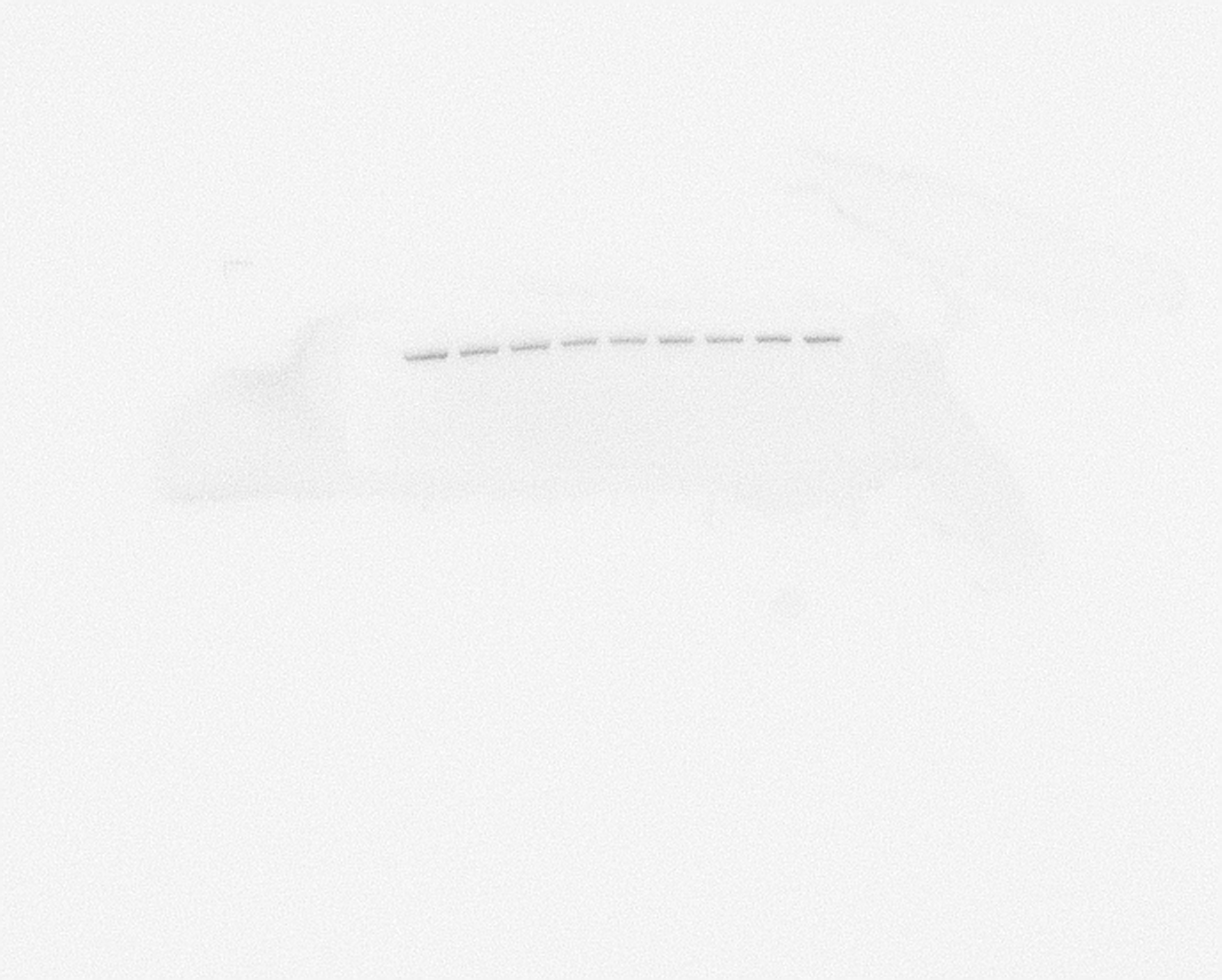

Supplement: Supplemental Information 3 [file peerj-11-15232-s003.zip › wb/RPS║═LINC/tubulin-du/3s│÷═╝.Tif]

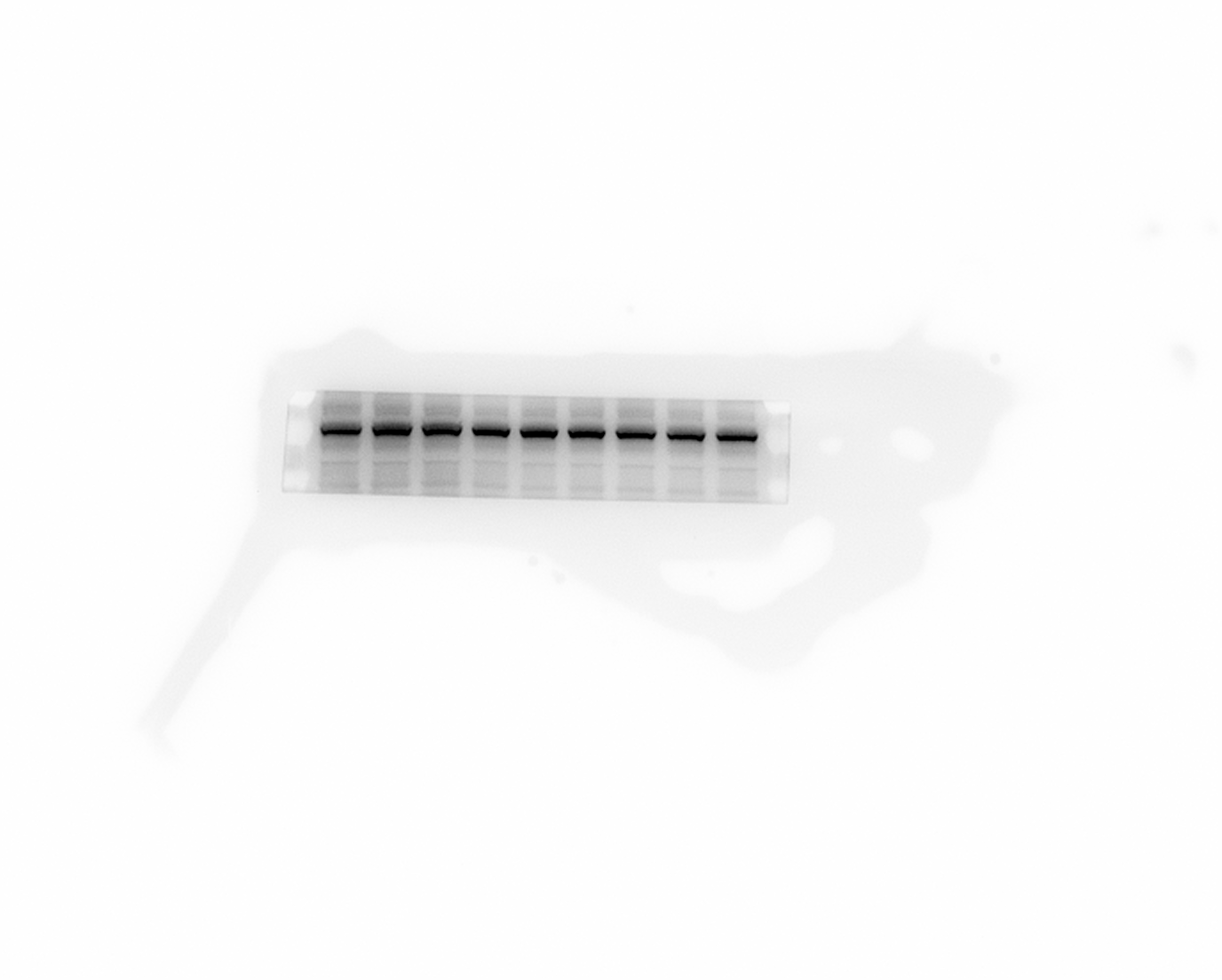

Supplement: Supplemental Information 3 [file peerj-11-15232-s003.zip › wb/RPS║═LINC/tubulin-pc/60s┐╔╙├.Tif]

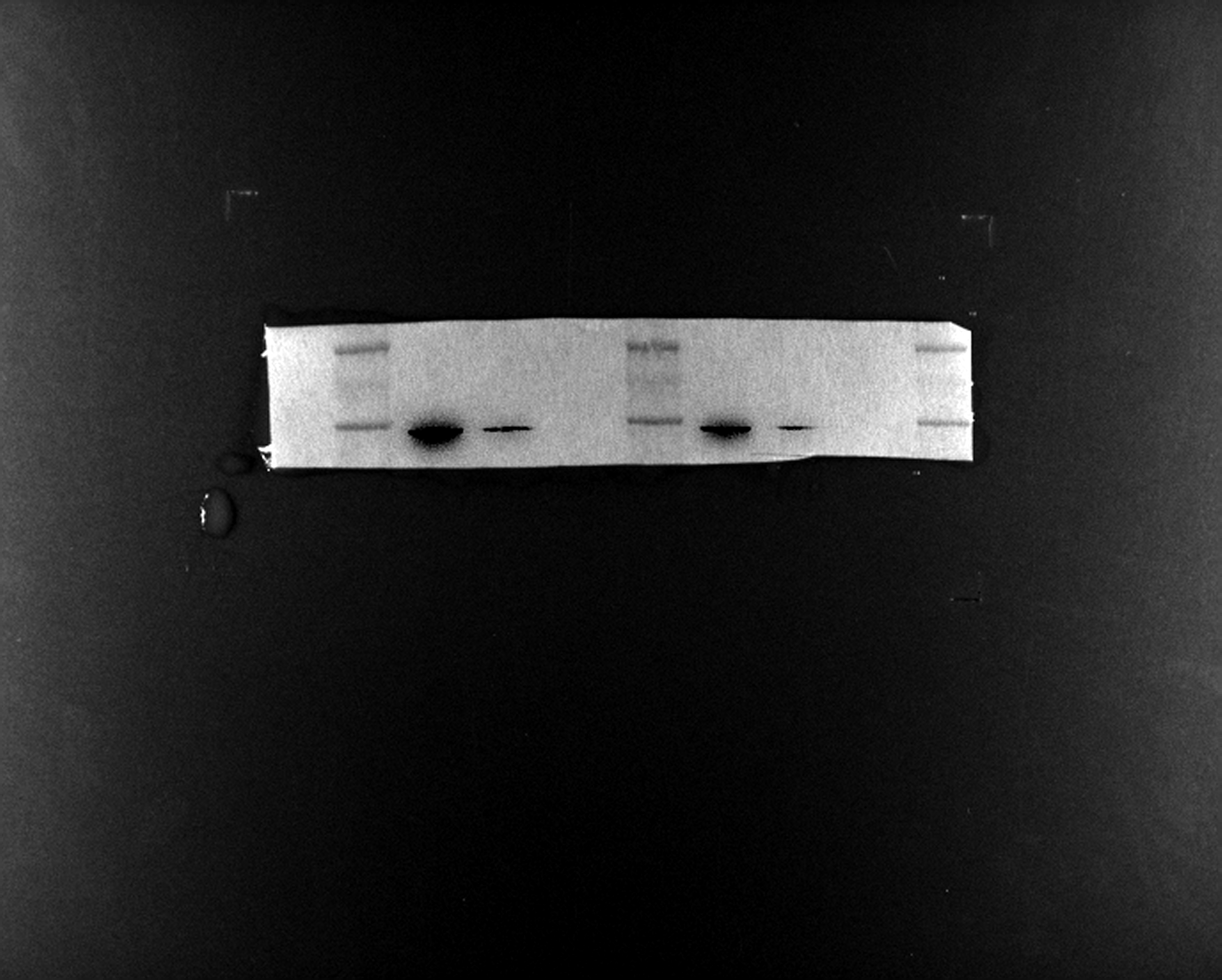

Supplement: Supplemental Information 3 [file peerj-11-15232-s003.zip › wb/pulldown/RPS19BP1/1+.tif]

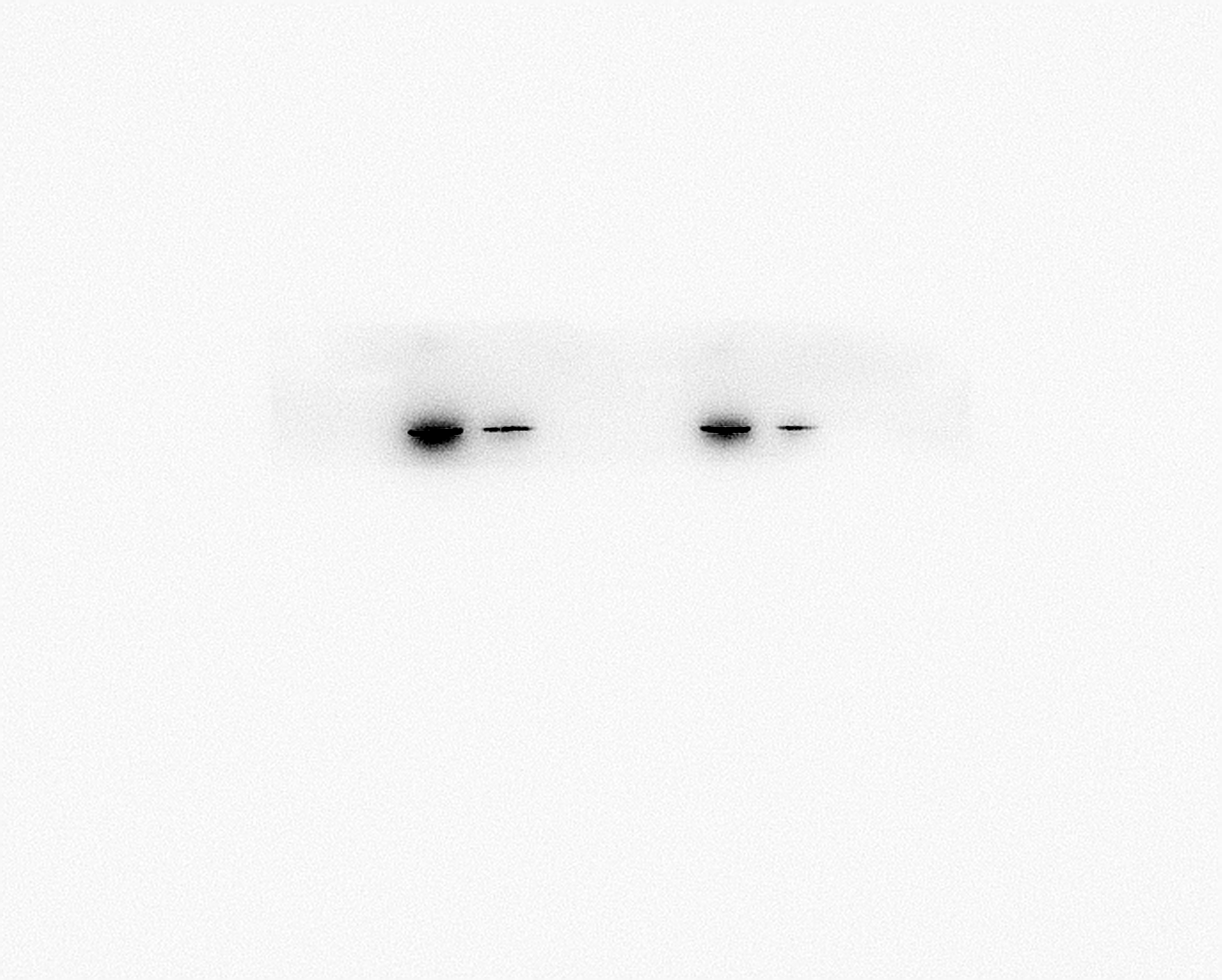

Supplement: Supplemental Information 3 [file peerj-11-15232-s003.zip › wb/pulldown/RPS19BP1/1.tif]

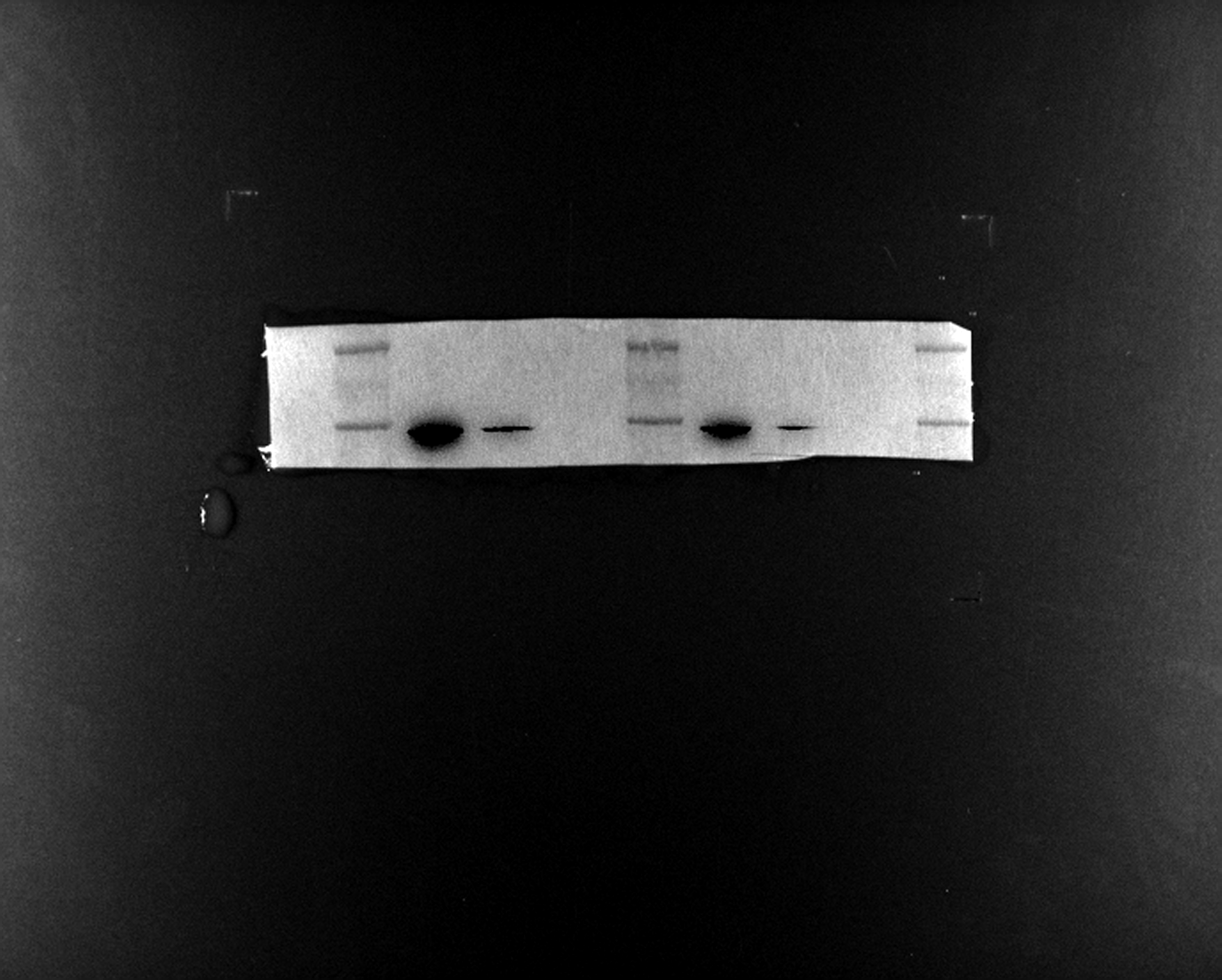

Supplement: Supplemental Information 3 [file peerj-11-15232-s003.zip › wb/pulldown/RPS19BP1/2+.tif]

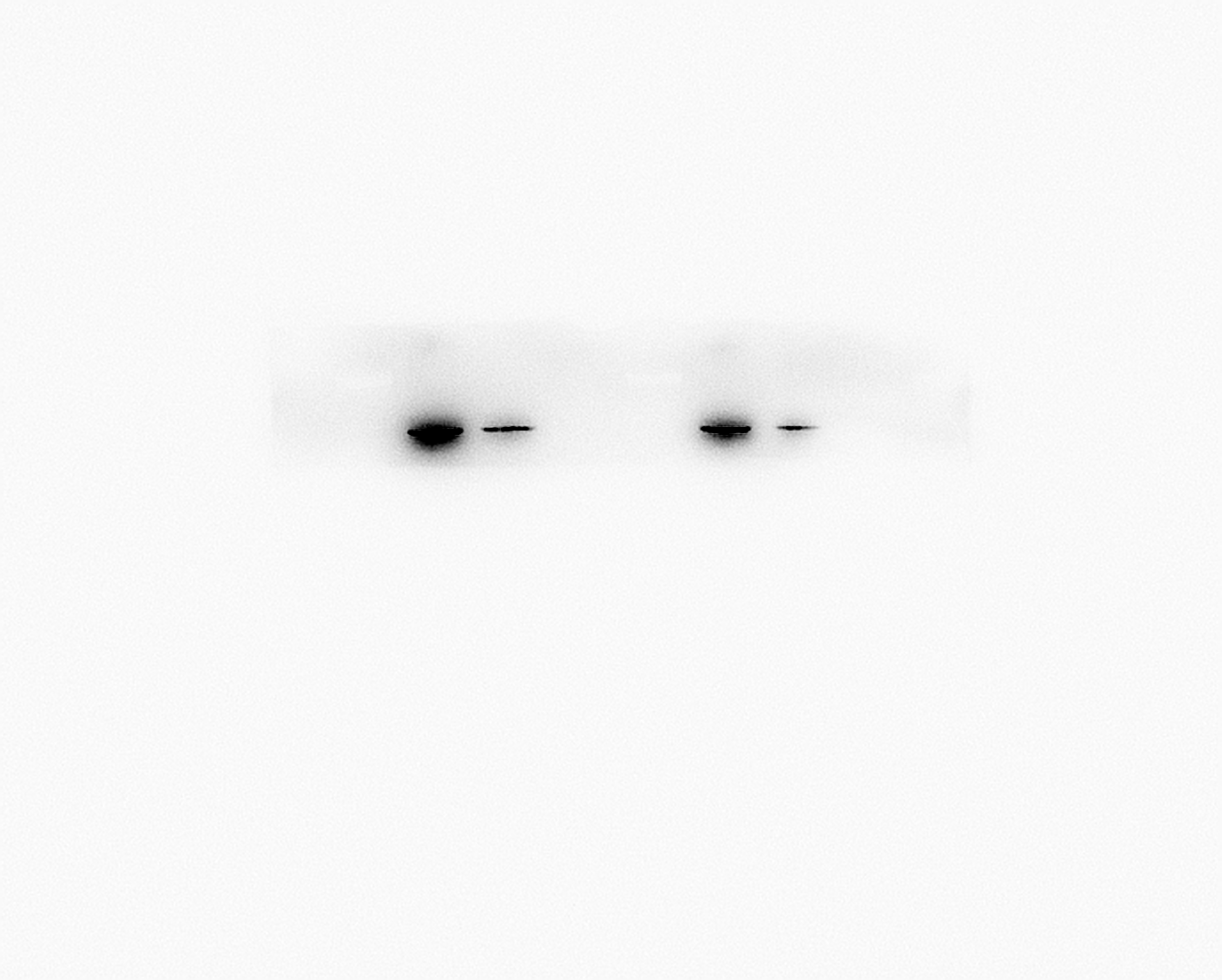

Supplement: Supplemental Information 3 [file peerj-11-15232-s003.zip › wb/pulldown/RPS19BP1/2.tif]

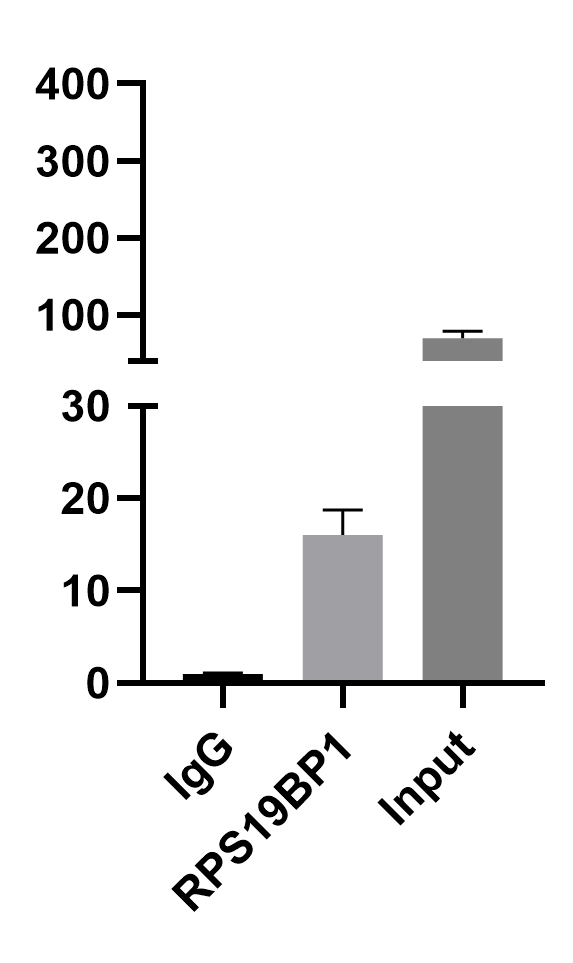

Supplement: Supplemental Information 3 [file peerj-11-15232-s003.zip › wb/pulldown/du.tif]

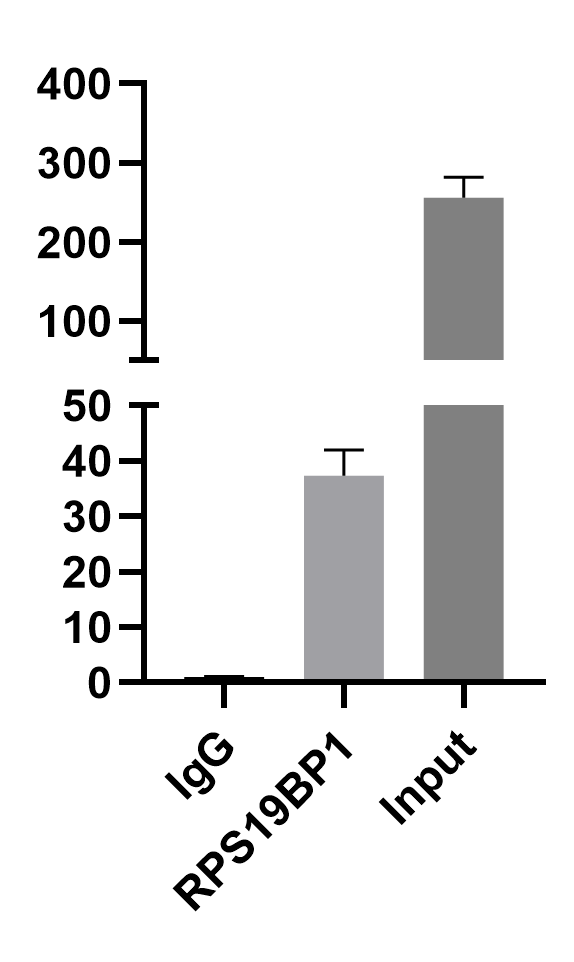

Supplement: Supplemental Information 3 [file peerj-11-15232-s003.zip › wb/pulldown/pc.tif]

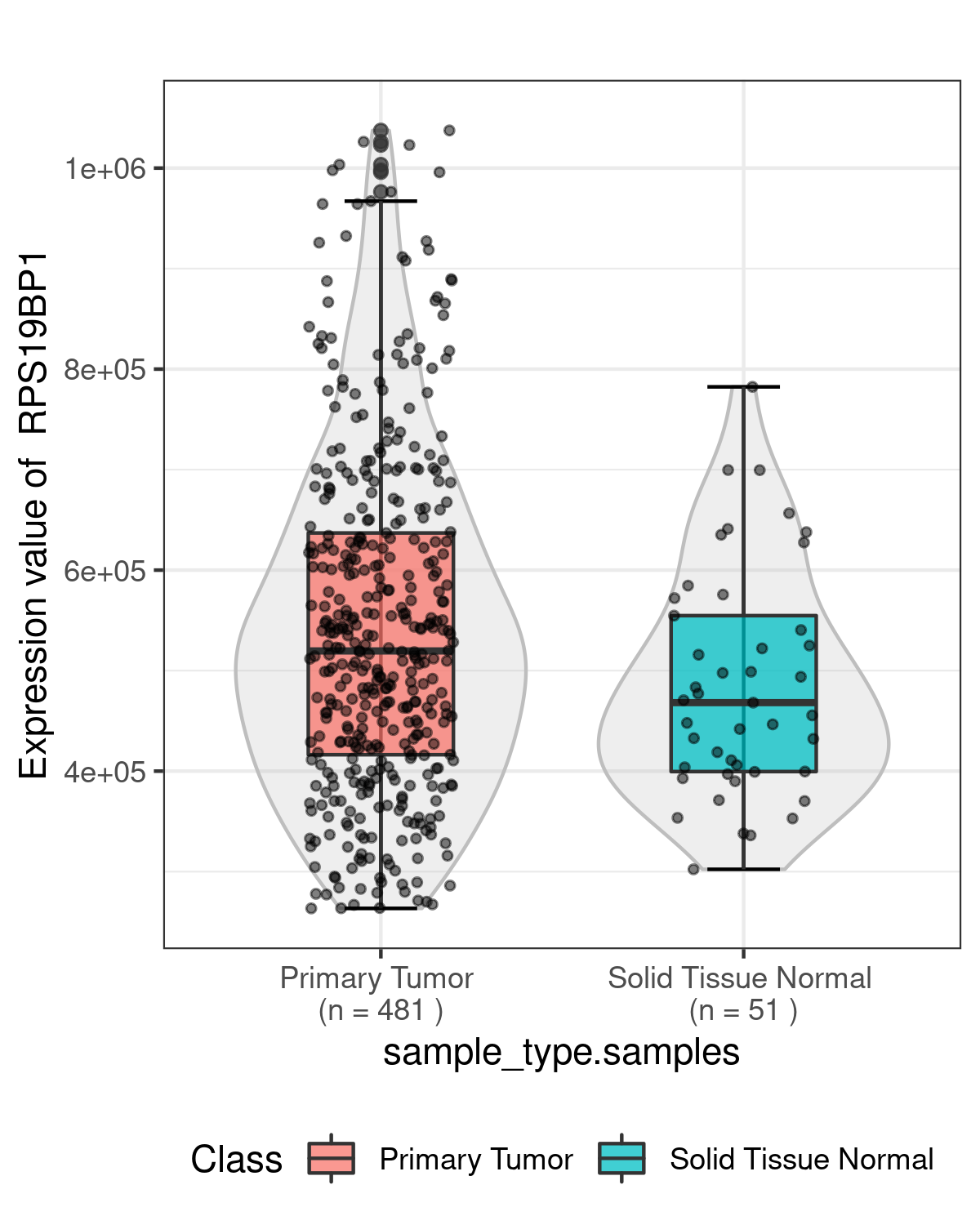

Supplement: Supplemental Information 3 [file peerj-11-15232-s003.zip › wb/pulldown/sample_type.samples.RPS19BP1.tiff]
